# Group B Streptococcus Bacteremia and Endocarditis in a Middle-Aged Male

**DOI:** 10.7759/cureus.20578

**Published:** 2021-12-21

**Authors:** Johnny M McKenzie, Lauren Pacheco

**Affiliations:** 1 Internal Medicine, Brookwood Baptist Medical Center, Birmingham, USA

**Keywords:** group b streptococcus (gbs), group b streptococcus agalactiae bacteremia, infective endocarditis, streptococcal bacteremia, mitral valve disease, valvular vegetation, conjunctival petechiae

## Abstract

Group B *Streptococcus* (GBS) is a rare but increasingly recognized cause of invasive disease in nonpregnant adults, particularly in the United States. Invasive GBS can take on many forms and may involve virtually any organ system. This case report describes the presentation, diagnosis, and management of a middle-aged male with GBS bacteremia and endocarditis.

A 59-year-old Caucasian male with a history of a heart murmur presented to the emergency department (ED) with two weeks of intermittent fevers, chills, rigors, and back pain. He had also become increasingly agitated and confused over this time. His heart murmur was discovered years prior during a work physical examination but was not investigated further. On arrival, he was afebrile but hypotensive and tachycardic. Physical examination revealed petechiae at the bilateral inferior palpebral conjunctivae as well as a grade 2 holosystolic murmur heard best at the apex. Abnormal laboratory findings included leukocytosis, transaminitis, elevated ferritin, and elevated D-dimer. Blood cultures were positive for *Streptococcus agalactiae*, and echocardiography demonstrated large mitral valve vegetations. The patient received intravenous (IV) antibiotics and eventually underwent a successful mitral valve replacement with a 31-mm pericardial tissue valve. No source of infection was identified in this patient despite an extensive workup.

The incidence of invasive GBS among nonpregnant adults has increased significantly in recent decades. The majority of affected patients are elderly and with significant underlying medical conditions. GBS bacteremia and endocarditis carry a very high mortality rate despite appropriate antimicrobial therapy. Combined medical-surgical therapy confers better outcomes in cases of endocarditis. Our patient's history of a heart murmur could have represented previously undiagnosed mitral valve pathology, placing him at higher risk of endocarditis. Apart from that, however, he lacked most of the typical risk factors associated with invasive GBS infections. Otherwise healthy patients with invasive GBS should undergo a comprehensive workup for potential underlying chronic illnesses. In the proper clinical context, conjunctival petechiae should elicit concern for infective endocarditis as they are present at a rate similar to that of Janeway lesions, splinter hemorrhages, and Roth spots.

## Introduction

*Streptococcus agalactiae*, also known as Group B Streptococcus (GBS), has long been a well-known cause of illness in neonates, infants, and pregnant women. Since surveillance studies conducted in the 1980s, however, GBS has been recognized as an increasingly common cause of invasive disease in nonpregnant adults. Previously, there were only sporadic reports involving this subgroup of the population. For unknown reasons, the incidence appears to be particularly high in the United States, with an estimated 11 cases per 100,000 in one 2016 analysis [1,2]. Nonpregnant adults are now estimated to represent greater than 75% of invasive GBS cases in the United States and around 90% of the mortality [3]. Invasive GBS can take on many forms including but not limited to abscesses, necrotizing fasciitis, osteomyelitis, septic arthritis, pneumonia, pyelonephritis, meningitis, endocarditis, and primary bacteremia without a focus. This case report describes the presentation, diagnosis, and successful management of a middle-aged male with GBS bacteremia and endocarditis.

## Case presentation

**Figure 1:**
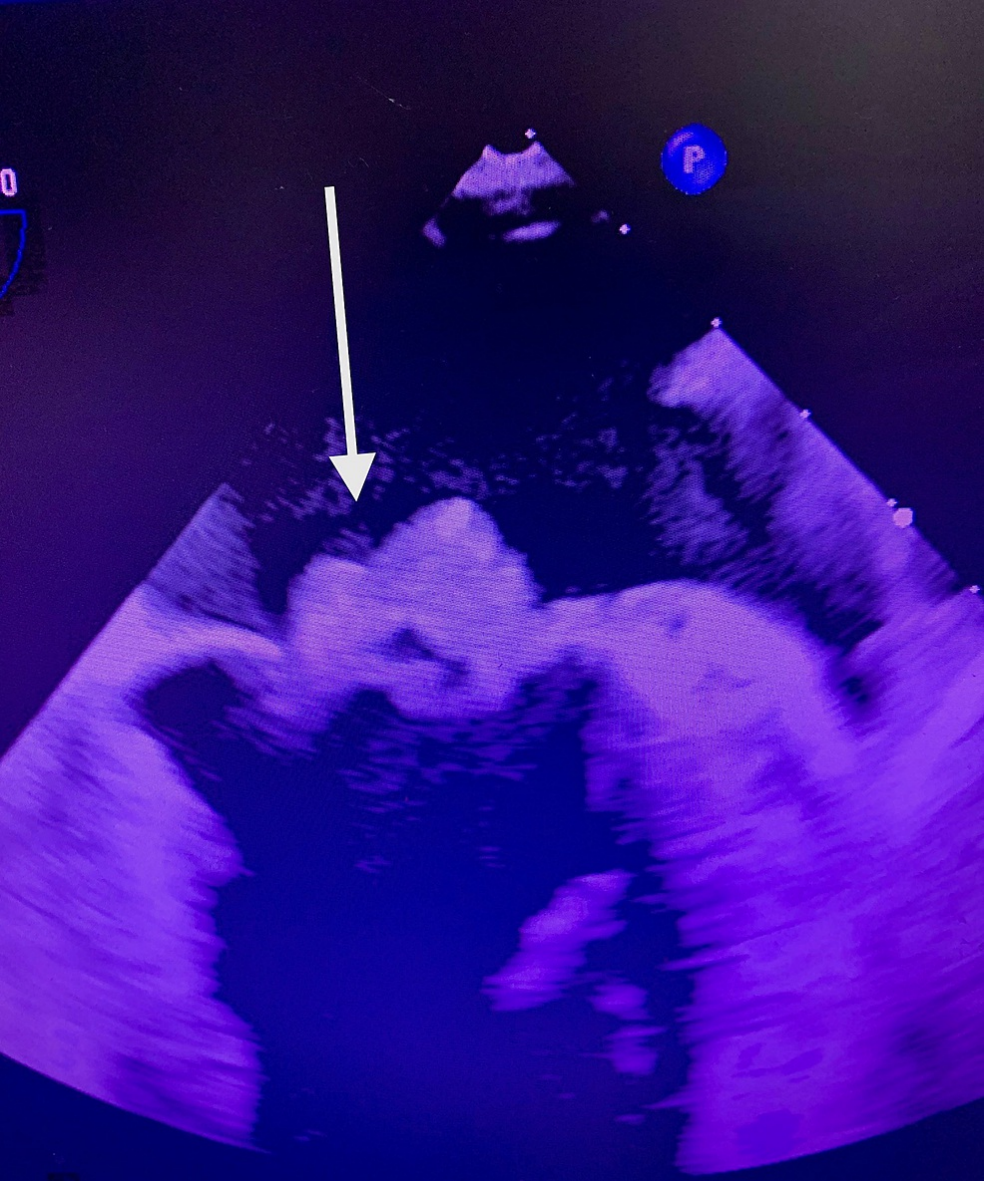
Large Mitral Valve Vegetations on Echocardiogram

**Figure 2:**
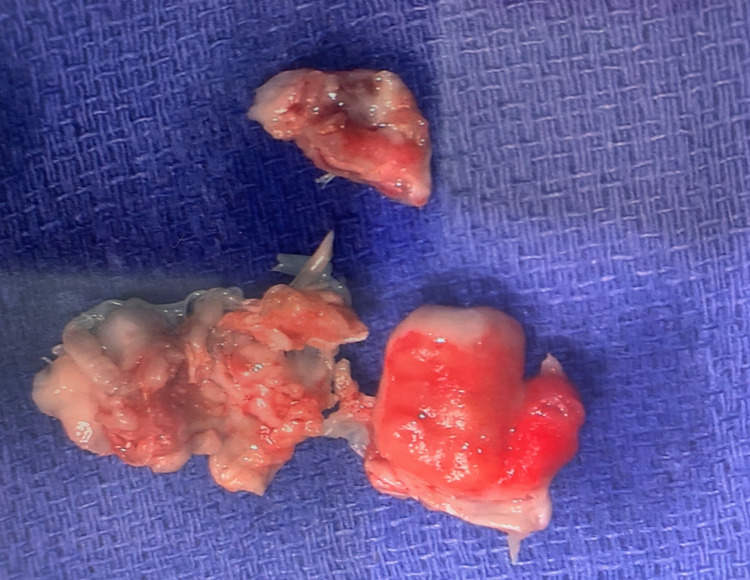
Resected Mitral Valve Leaflets and Vegetations

A 59-year-old Caucasian male with a history of a heart murmur presented to the emergency department (ED) on Christmas Eve with two weeks of intermittent fevers, chills, rigors, and back pain. A few days prior, he visited a different ED with similar symptoms and was discharged home with nitrofurantoin for a presumed urinary tract infection (UTI). His symptoms got worse, however, and his wife stated that he had become increasingly agitated and confused over this time. This involved erratic behavior and random episodes of anger outbursts, which were uncharacteristic of him. He reported no sick contacts or recent hospitalizations. He denied any associated symptoms including headache, numbness, weakness, neck stiffness, chest pain, palpitations, cough, dyspnea, abdominal pain, vomiting, diarrhea, and dysuria. He had received no routine medical care for most of his adult life outside of the occasional employment physical examination. A heart murmur was discovered at one of these physical examinations several years prior but was not investigated further as it was felt to be nonpathological. Otherwise, he had always received a clean bill of health. He was taking no medications except for nitrofurantoin, which he was scheduled to complete the next day. He denied alcohol and tobacco use. He reported no current or past intravenous (IV) drug use. He was born in the United States and had never traveled elsewhere. On presentation, he was hypotensive at 94/55 mmHg, tachycardic at 101 beats/minute, and afebrile at 98.4°F. He was alert and oriented but easily distractible and with a disorganized thought process, which his wife confirmed was abnormal. Cardiac auscultation revealed a grade 2 holosystolic murmur heard best at the apex. The neurological examination was unremarkable. His back pain was reproduced with movement, and there was no tenderness to palpation at the spine or paraspinal muscles. Scattered petechiae were noted at the bilateral inferior palpebral conjunctivae. There were no Osler nodes, Janeway lesions, or splinter hemorrhages. Abnormal laboratory findings included elevations in white blood cell (WBC) count at 11,600/mcL (81.7% neutrophils), aspartate aminotransferase at 122 unit/L, alanine transferase at 158 unit/L, ferritin at 2,130 ng/mL, lactate dehydrogenase at 350 unit/L, and D-dimer at 22.24 mcg/mL. Urinalysis showed 11-20 WBC/high-powered field but was negative for nitrite and leukocyte esterase. Serum creatinine was normal at 0.87 mg/dL. Chest X-ray and brain non-contrast CT were both without abnormalities. Blood cultures were drawn prior to the initiation of IV vancomycin and ceftriaxone, and the patient was admitted to the floor. His blood pressure and heart rate normalized with IV normal saline. His transaminitis and leukocytosis resolved by the following morning. On day three of admission, all blood cultures were positive for *Streptococcus agalactiae*, while his urine culture remained negative. IV ceftriaxone was continued alone at 2 g daily. Transthoracic echocardiogram was delayed by a few days due to limited hospital staffing over Christmas week but ultimately revealed large mitral valve vegetation with moderate to severe mitral regurgitation. Transesophageal echocardiogram (TEE) was then obtained and showed a 2.1 × 1.5 cm vegetation on the anterior leaflet in addition to a 2.5 × 0.5 cm vegetation on the posterior leaflet of the mitral valve (Figure 1). The patient elected to forgo imaging of his spine despite ongoing back pain. Six days after the TEE, he underwent a successful mitral valve replacement with a 31-mm pericardial tissue valve via right thoracotomy (Figure 2). Resolution of the bacteremia was confirmed with repeat blood cultures, and the patient was discharged in stable condition four days postoperatively to complete a six-week course of IV ceftriaxone via home health services.

## Discussion

In the United States, the incidence of invasive GBS among nonpregnant adults has increased in recent years despite a steady decline of infections seen in neonates and pregnant women. The vast majority of cases occur in patients with at least one underlying condition, the most common of which are uncontrolled diabetes mellitus and obesity [2,4]. Other associated conditions include malignancy, HIV, and advanced renal and liver disease [5,6]. The risk is especially high in the elderly, particularly among those who reside in nursing homes [5,7].

Primary bacteremias with no known source and endocarditis comprise around 30% and 2%-9% of invasive GBS cases, respectively [5,8]. As demonstrated in our patient, GBS endocarditis typically features large vegetations involving native left-sided valves. GBS causes a particularly virulent form of streptococcal endocarditis, carrying mortality of up to 40% in both acute and subacute cases despite appropriate antimicrobial therapy [9]. There is evidence that combined medical-surgical therapy yields better outcomes in these patients [10].

Like many of those with GBS bacteremia, no definitive source was identified in our patient despite an extensive workup. He also lacked typical risk factors such as diabetes, HIV, chronic kidney or liver disease, and obesity. Underlying malignancy was not completely ruled out during this admission, however, and he was strongly encouraged to undergo colon cancer screening as an outpatient. Given his preexisting murmur, he certainly could have had underlying mitral valve pathology, rendering him more susceptible to infective endocarditis, but we are unable to confirm this.

The presence of conjunctival petechiae in this patient is also worthy of discussion. Petechiae, both mucosal and dermatologic, can serve as important clues for infective endocarditis. They are present in about 5% of cases, a rate similar to that of the more often discussed Janeway lesions, splinter hemorrhages, and Roth spots [11]. Therefore, it is important to perform a thorough ocular examination, including visualization of the palpebral conjunctiva, in patients suspected of having endocarditis.

## Conclusions

Group B Streptococcus (GBS) has emerged as an increasingly common cause of serious illness in nonpregnant adults. Invasive GBS can take on many different forms including bacteremia without a source and endocarditis, each carrying a very high mortality rate. The risk of invasive disease is especially high in the elderly, and the vast majority of affected patients have at least one underlying medical condition. Invasive GBS in otherwise healthy patients should prompt a comprehensive workup for potential underlying illnesses. In the proper clinical context, the presence of conjunctival petechiae should elicit concern for infective endocarditis.
